# Development of an IP-Free Biotechnology Platform for Constitutive Production of HPV16 L1 Capsid Protein Using the *Pichia pastoris PGK1* Promoter

**DOI:** 10.1155/2015/594120

**Published:** 2015-05-18

**Authors:** F. C. Mariz, E. C. Coimbra, A. L. S. Jesus, L. M. Nascimento, F. A. G. Torres, A. C. Freitas

**Affiliations:** ^1^Laboratorio de Estudos Moleculares e Terapia Experimental, Departamento de Genética, Universidade Federal de Pernambuco, 50670-901 Recife, PE, Brazil; ^2^Departamento de Microbiologia, Centro de Pesquisas Aggeu Magalhaes, 50740-465 Recife, PE, Brazil; ^3^Laboratorio de Biologia Molecular, Departamento de Biologia Celular, Universidade de Brasília, 70910-900 Brasília, DF, Brazil

## Abstract

The human papillomavirus (HPV) L1 major capsid protein, which forms the basis of the currently available vaccines against cervical cancer, self-assembles into virus-like particles (VLPs) when expressed heterologously. We report the development of a biotechnology platform for HPV16 L1 protein expression based on the constitutive *PGK1* promoter (P_PGK1_) from the methylotrophic yeast *Pichia pastoris*. The L1 gene was cloned under regulation of P_PGK1_ into pPGKΔ3 expression vector to achieve intracellular expression. In parallel, secretion of the L1 protein was obtained through the use of an alternative vector called pPGKΔ3*α*, in which a codon optimized *α*-factor signal sequence was inserted. We devised a work-flow based on the detection of the L1 protein by dot blot, colony blot, and western blot to classify the positive clones. Finally, intracellular HPV VLPs assembly was demonstrated for the first time in yeast cells. This study opens up perspectives for the establishment of an innovative platform for the production of HPV VLPs or other viral antigens for vaccination purposes, based on constitutive expression in *P. pastoris*.

## 1. Introduction

HPVs are a large family of dsDNA viruses that cause benign warts and malignant tumors. Persistent infection by HPV imposes a huge burden on health services worldwide owing to its links with cancer of the vagina, vulva, penis, anus, tongue, and, in particular, uterine cervix, which is the most serious outcome [1]. About 75% of sexually active people are exposed to HPV during their lifetime [2]. Some of the nearly 120 HPV genotypes reported [3] are encountered in virtually 100% of cervical tumors and can thus be classified as high-risk HPV (hr-HPV) types [4]. Nearly 1.4 million women are affected by cervical cancer all over the world while 520,000 new cases and 274,000 resulting deaths are reported annually, which leads to a mortality rate of 55% [5]. Although HPV16 and HPV18 are responsible for 70% of cervical cancer cases worldwide [5], there are other 12 hr-HPV types whose prevalence is subject to regional variations [6]. In Brazil, HPV16 is the most common genotype, but HPV31 and HPV33 are as prevalent as HPV18, at least in the Northeast and Midwest Regions [7], which illustrates the urgent need to set up vaccination programmes where HPV is prevalent. Apart from the cervical intraepithelial lesions of all grades and warts, it is estimated that 5% of all human cancers are associated with this viral infection [8].

Middle-income developing countries, where more than 80% of the related deaths are found, have failed to establish cervical screening programs in a satisfactory manner [5]. Since 2008, two protective vaccines have been licensed for prophylaxis against HPV infection. Gardasil (Merck) and Cervarix (GlaxoSmithKline) contain HPV VLPs produced in* Saccharomyces cerevisiae* and baculovirus-infected cells, respectively. Although these vaccines are highly effective and safe [9, 10], their prohibitive costs prevent them from being widely available in developing countries [11]. However, the regional production of prophylactic HPV vaccine could overcome the problem of this price barrier by reducing the cost and also filling the current demand and supply gap [12].

Production of HPV VLPs can be achieved through the expression of recombinant major capsid protein L1 in heterologous systems [14, 15]. These particles preserve the conformational epitopes from native virions and are thus able to induce high titers of neutralizing antibodies [14]. Different expression platforms have been explored for producing HPV VLPs with varying degrees of success [14, 16–20]. Bacterial expression systems are limited to producing economically significant amounts of recombinant HPV VLPs [20, 21] and, among the eukaryotic systems, yeast cells have the greatest potential because of their high expression levels, combined with simple growth requirements and high growth rates.

The expression and characterization of HPV VLPs from the methylotrophic yeast* Pichia pastoris* has been described elsewhere [12, 22–24]. In these studies, the expression of HPV L1 genes was under the control of the promoter from the alcohol oxidase I gene (*AOX1*), which is tightly regulated at the transcription level. Recombinant protein expression under the control of P_AOX1_ relies on a preliminary production of yeast biomass through cultivation on glucose/glycerol, followed by induction of protein production in the presence of methanol as the sole carbon source [25]. Despite the success of the AOX1-based system, the use of methanol as an inducer has drawbacks such as its inflammability, toxicity, and biomass generation requirements prior to the induction phase. This means there is the need for longer time-based protocols, a rigid control of methanol levels during the induction phase, and the use of an inducer compound, which is particularly unsuitable when planning an industrial platform [25]. Furthermore, there is evidence that the culture conditions required for P_AOX1_ induction can compromise the expression levels of other VLPs and this can affect HPV L1 expression [22, 26, 27].

The isolation and molecular characterization of the 3-phosphoglyceratekinase gene (*PGK1*) from* P. pastoris* was reported by de Almeida et al. [28]. In yeast,* PGK1 *encodes a glycolytic enzyme which also acts in the gluconeogenic pathway and may represent 5% of the total cellular protein [29]. Secretion of* Bacillus subtilis α*-amylase protein was carried out effectively under the control of the constitutive P_PGK1_ from* P. pastoris* cells grown in glucose, glycerol, or methanol, whereas cells grown in glucose displayed higher expression levels [28]. Unlike the P_AOX1_-based system, biomass generation and protein production occur simultaneously in medium containing glucose or glycerol. Although a constitutive expression is not recommended when the protein of interest is toxic to the yeast cell [26, 30], this is not the case for HPV L1 protein since its expression has been efficiently achieved for approximately 144 hours [31]. These features make the P_PGK1_ an attractive system for heterologous expression in* P. pastoris*.

In this study, we explored the development of an innovative heterologous expression system for production of HPV16 L1 protein. For this purpose, the P_PGK1_-driven constitutive expression of the L1 protein was investigated, both through intracellular and secretory pathways. In order to select well-expressing yeast clones, we combined the use of dot blot, colony blot, and western blot techniques in a workflow for the detection of the L1 protein. Additionally, evidence that HPV L1 protein self-assembles into VLPs was observed* in vivo* by transmission electron microscopy. To date, this is the first report of heterologous expression* in P. pastoris *which uses the P_PGK1_ for biopharmaceutical purposes.

## 2. Materials and Methods

### 2.1. Strains and Cell Culture Media

*Escherichia coli* DH5*α* strain [F Φ80*lac*ZΔM15 Δ(*lac*ZYA-* arg*F)U169* rec*A1* end*A1* hsd*R17(rk−, mK+)* pho*A supE44 *λ*-* thi-*1* gyr*A96*rel*A1] was routinely used as a host for cloning and plasmid manipulations. This strain was cultured at 37°C in LB medium (0.5% yeast extract, 1% NaCl, 1% tryptone) supplied with appropriate antibiotics.

The* P. pastoris* X-33 strain (wild-type) used in this study was purchased from Invitrogen. The yeast cells were grown at 30°C on YPD medium (1% yeast extract, 2% Bacto-peptone, 2% glucose) and YPDS (1% yeast extract, 2% peptone, 2% glucose, 1 M sorbitol) supplemented with 100 *μ*g/mL zeocin (Invitrogen) when necessary.

All the molecular cloning techniques were carried out as previously described [32]. Restriction enzymes used for cloning were purchased from Promega and used in accordance with manufacturer's recommendations. DNA sequencing analysis was performed on a Genetic Analyser 3500 automatic sequencer (Life Technologies).

### 2.2. Cloning of the HPV16 L1 and Construction of Expression Vectors

A DNA sequence encoding the HPV16 L1 protein (Gen Bank access number GI: 27752860) was designed with codons optimized for expression in* P. pastoris*. Restriction sites for* Xho*I and* Not*I were added to the flanking regions of the L1 gene so that the cloning could be directed into the expression vector. The gene was synthesized by Epoch Biosciences (TX, USA), and cloned into pBSK plasmid. The resulting construct called pBSK/L1 was amplified in* E. coli* DH5*α*. L1 gene was released from pBSK after double-digestion with* Xho*I and* Not*I and employed for creating the expression vectors.

Two expression vectors were constructed with the P_GK1_ promoter from* P. pastoris *for constitutive expression of the L1 protein. The original 2 kb P_PGK1_ sequence described by de Almeida et al. [28] was reduced to a minimal ~400 bp sequence after deletion analysis with restriction enzymes [33]. The resulting P_PGK1_ sequence was used to generate the pPGKΔ3 expression vector for intracellular expression. Additionally, a pPGKΔ3*α* expression vector carries a codon-optimized* S. cerevisiaeα*-factor signal sequence (*α*-MF) cloned downstream to P_PGK1_ to drive recombinant protein secretion. Both P_PGK1_-based vectors contain the zeocin resistance gene* Sh ble* for positive selection of* E. coli* and* P. pastoris *recombinants, as well as a C-terminal polyhistidine (6xHis) tag for detection of the fusion protein by immunoblot assays. The L1 gene previously digested with* Xho*I and* Not*I was cloned into pPGKΔ3*α* and pPGKΔ3 expression vectors digested with the same enzymes, and the resulting vectors were called pPGKΔ3*α*/L1 and pPGKΔ3/L1, respectively. The construction of the vectors was confirmed by restriction digestion, PCR and DNA sequencing with specific primers for both L1 (5′ TAGGATCCATGTCATTATGGCTTCCA 3′ and 5′ CTGGATCCTTAATGATGATGATGATGATGCAA 3′ which flank the entire gene while 5′ GGTCAACCTTTAGGAGTTGG 3′ and 5′ GACGAACATTTGTTCCCTTCA 3′ amplify an internal L1 fragment of ~400 bp) and P_PGK1_ (5′ TCATAGTTCATCCCTCTCTCC 3′) sequences.

### 2.3. Electroporation of Yeasts and Selection of* P. pastoris* Recombinant Strains

Stable integration into the* PGK1* locus of* P. pastoris *was achieved after linearization of the 5 *μ*g P_PGK1_-based expression vectors with* Sac*I [33].* P. pastoris *electrocompetent cells were prepared and transformed, as described elsewhere [13]. In this work, yeast clones were either referred as* P. pastoris*/pPGKΔ3*α*/L1 or* P. pastoris*/pPGKΔ3/L1, depending on the vector that was used.

After selection on agar plates containing YPD supplemented with 100 *μ*g/mL zeocin, the transformants were subjected to a general procedure for the selection of clones containing multiple copies of the expression cassette, called Posttransformational Vector Amplification (PTVA) process [13]. For this purpose, yeast clones were later plated on higher concentrations of zeocin (100, 500, and 1000 *μ*g/mL).

### 2.4. Screening of Well-Expressing* Pichia* Clones by Dot Blot and Colony Blot Assays

Multicopy clones resistant to 1000 *μ*g/mL zeocin were subjected to a general screening for selection of recombinant strains expressing high levels of HPV L1 protein. The screening was carried out as follows: (i)* P. pastoris*/pPGKΔ3*α*/L1 clones were cultivated in agar plates and subsequently subjected to colony blot for detection of HPV L1 protein with the CamVir anti-HPV16 L1 monoclonal antibody (Chemicon, USA); (ii) In contrast,* P. pastoris*/pPGKΔ3/L1 clones were subsequently grown in deep-well plates to detect HPV L1 protein by dot blot with the anti-L1 monoclonal antibody.

In the case of colony blot, the protocol described by Goodnough et al. [34] was modified as follows. After cultivation of* P. pastoris*/pPGKΔ3*α*/L1 multicopy clones in YPD agar plates for 3 days at 30°C, the PVDF membranes were cut as discs and left standing with the surface colonies for 3 hours at 28°C. Colonies on the master plate could be replicated by placing the PVDF discs on a fresh agar plate when desired. The PVDF discs were washed with tris-buffered saline (TBS) containing 0.05% Tween 20 (TBST) to remove adhering cells. The membranes were blocked by incubation in TBST, supplemented with 5% nonfat milk for 1 hour at room temperature, and then incubated overnight at 4°C with anti-L1 antibody properly diluted (1 : 1000) in the blocking solution. PVDF discs were washed three times with TBST for 10 min and then incubated for 1 hour at room temperature with peroxidase-conjugated goat anti-mouse immunoglobulin (IgG, Sigma-Aldrich) diluted 1 : 3000 in the blocking solution. Chemiluminescence reaction was detected with an ECL kit (GE Healthcare).

For the dot blot assay,* P. pastoris*/pPGKΔ3/L1 multicopy clones were first grown in a deep-well plate containing YPD medium for 3 days at 30°C. The plate was shaken at 300 rpm and* P. pastoris* cells were harvested by centrifugation at 3000 rpm for recovery of the pellets. Preparation of yeast extracts was performed with an “alkaline lysis” procedure as previously described [35]. Briefly, the cell pellets were resuspended in lysis buffer (0.1 M NaOH, 0.05 M EDTA, 2% SDS, 2% *β*-mercaptoethanol) and heated to 90°C for 10 min and the lysate was brought to neutral pH. To improve solubilization, the lysate was heated again for 10 min and mixed with loading buffer (0.25 M Tris-HCl pH 6.8, 50% glycerol). The PVDF membranes were cut in a proper way and inserted into a dot blotter apparatus. Protein transfer to the membranes was carried out with 100 *μ*L of the lysate for 1 hour and, thereafter, the immunoblot proceeded as already described for the colony blot. The HPV16 L1 protein that was episomally expressed in* P. pastoris *[22] was used as positive control for both dot blot and western blot assays.

### 2.5. Protein Expression in Shake-Flask Cultures

*P. pastoris* multicopy clones were selected for baffled-flask cultivation in accordance with the highest detected level for the HPV L1 protein.* P. pastoris*/pPGKΔ3*α*/L1 clones were first preinoculated in 5 mL of YPD to achieve the secretion of the L1 protein and grown at 28–30°C in a shaking incubator (250–300 rpm) until the cultures reached an OD_600_ of ~10 (24 hours). Following this, the cells were inoculated in 20 mL of YPD for 3 days and aliquots were taken for analysis at periodic intervals. These samples were centrifuged at 12000 g for 2 min and the supernatants were stored for further analysis. For the intracellular expression of the L1 protein,* P. pastoris*/pPGKΔ3/L1 clones were subjected to the same protocol but the cell pellets were stored instead of the supernatants.

### 2.6. Preparation of Intra- and Extracellular Protein Extracts

Aliquots of supernatants were submitted to precipitation with trichloroacetic acid (TCA) (Sigma-Aldrich) to a final concentration of 10% to allow the secretory production of the HPV L1 protein to be analyzed. After concentration, the supernatant was discarded, and the protein pellet was resuspended again in 100% acetone to remove residual TCA. A final volume of SDS-loading buffer (1 M Tris-HCl, pH 6.8, 10% SDS, 0.5% *β*-mercaptoethanol, 0.1% bromophenol blue) was then added to the washed pellet (corresponding to 100x concentration) and this mix was resolved on 15% SDS PAGE after the samples had been heated for 10 min at 75°C. Polyacrylamide gel was either stained with Coomassie brilliant blue (Pierce, IL, USA) or transferred to PVDF membranes using a V20-SDB semidry protein transfer apparatus (Scie-plas, Cambridge, UK). Immunoblot was performed as described in Section 2.4.

Preparation of cell extracts for intracellular analysis of the L1 expression was carried out with breaking buffer and acid-washed sterile glass beads (0.45 mm in diameter), according to Cregg et al. [36]. This crude protein sample was mixed in gel loading buffer and prepared for SDS PAGE and western blot (as described above).

### 2.7. Electron Microscopy

At the end of the induction course, both the cells and culture supernatants were subjected to absorption into carbon-coated grids, as recommended by Falcón et al. [37]. The grids were subjected to examination with a FEI Morgani 268D transmission electron microscope, operated at 100 kV, in order to analyze the VLP formation in cytoplasm and culture medium of recombinant yeast-expressing HPV L1 protein.

## 3. Results and Discussion

### 3.1. Construction of Expression Vectors and Generation of* Pichia* Recombinant Strains

**Figure 1:**
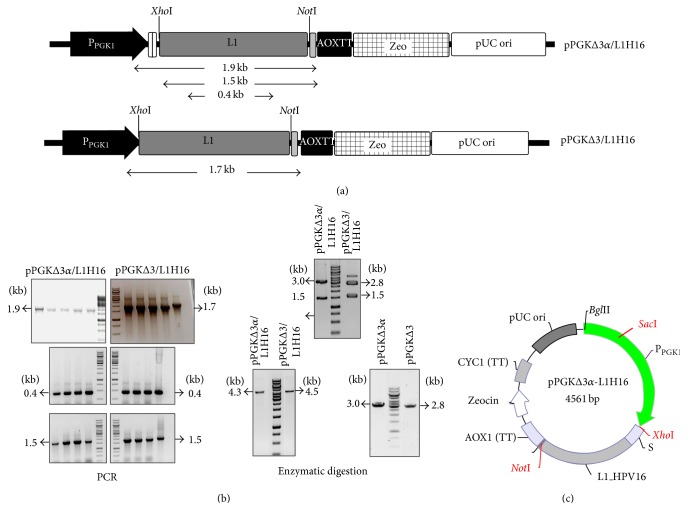
Construction of cassettes for HPV16 L1 gene expression under the control of P_PGK1_. (a) Schematic illustration of the two expression cassettes in which fragment lengths are highlighted for the confirmation analysis: primers flanking part of the P_PGK1_ and the L1 gene render a 1.9 kb fragment from the pPGKΔ3*α*/L1H16 vector and a 1.7 kb fragment from the pPGKΔ3/L1H16 vector; primers flanking the L1 gene renders a 1.5 kb fragment from both vectors; internal L1 primers render a 0.4 kb fragment from both vectors. (b, left panel) Expression vectors were extracted from recombinant bacterial strains and subjected to PCR analysis: the three DNA fragments predicted in (a) were amplified from the extracted DNA plasmids. (b, right panel) Extracted DNA plasmids were further confirmed by restriction digestion with* Xho*I and* Not*I enzymes (upper line), through which the release of the L1 gene (1.5 kb) could be observed, along with the presence of pPGKΔ3*α* (3 kb) and pPGKΔ3 (2.8 kb) vectors. After confirmation by PCR and restriction digestion, the P_PGK1_-based cassettes were linearized with* Sac*I (lower line) prior to transformation of* P. pastoris*. (c) Map of pPGKΔ3*α*-L1H16.* PGK1* promoter and* AOX1* transcription terminator regions are flanking the L1 gene at its 5′ and 3′ ends, respectively. Besides the* E. coli* pUC origin and zeocin selection maker, this construct carries a codon-optimized* S. cerevisiaeα*-MF (S) downstream from the* PGK1* promoter, which is absent in the pPGKΔ3/L1H16 vector. The positions of* Xho*I,* Not*I, and* Sac*I restriction sites are also highlighted on the map. The map is merely illustrative and there is no correlation between the sizes of the highlighted regions.

For the production of HPV16 L1 protein, we used a heterologous expression system based on the constitutive* P. pastoris PGK1* promoter which was originally described and employed for secretion of *α*-amylase from* Bacillus subtilis *[28]. In this work, a variant of the* PGK1 *promoter sequence containing ~400 bp was used to allow integration of the expression vector via homologous recombination, as well as a codon-optimized *α*-factor from* S. cerevisiae* to drive the secretion of recombinant proteins (Figures 1(a) and 1(c)). Since previous studies have analyzed different protocols to optimize VLP production and purification steps in yeast [38–41], we believe that secretion of HPV L1 in the culture media could assist downstream processing. The analysis by PCR, DNA sequencing (data not shown), and restriction digestion showed the successful cloning of a codon-optimized L1 gene into P_PGK1_-based vectors (Figure 1(b), left panel).* P. pastoris*/pPGKΔ3*α*-L1H16 and* P. pastoris*/pPGKΔ3-L1H16 recombinant strains were obtained after electroporation of yeast cells with the linearized expression cassettes (Figure 1(b), right panel) and further selection on agar plates containing zeocin.

Although recombination with the expression cassette confers zeocin-resistance to* P. pastoris *cells, there is no way to ensure that a heterologous gene will be expressed at high levels. The selection of clones containing multiple copies of an expression cassette represents an attractive strategy for increasing expression levels [13]. Multicopy clones can be screened by transformants that are resistant to high levels of a selectable marker compound. However, this effective method is still laborious and inefficient, since 50 to 100 transformants usually need to be screened to have a reasonable chance of finding the 1-2% multicopy (>10 copies) clones [42]. Recently, an iterative process termed Posttransformational Vector Amplification (PTVA) has been investigated to generate* P. pastoris* clones containing multiple copies of the entire vector in the genome through the submission of transformants, which were initially selected on a low level of drug and only contained one or a few copies of the vector, to higher levels of zeocin [13]. Molecular details of this process are still unknown, but an analysis of PTVA-selected clones showed a three-to-five-fold increase in the vector copy number, as well as the integration of all the copies into the* P. pastoris *genome in the same* locus* as the original copy. In this work, we tested the generation of multicopy clones by the PTVA process starting with 55 transformants from each cassette. After growth on agar plates with increasing zeocin levels, 50* P. pastoris*/pPGKΔ3-L1H16 clones and 52* P. pastoris*/pPGKΔ3*α*-L1H16 clones showed resistance to 1000 *μ*g/mL zeocin. Zeocin resistance levels can be directly correlated with the copy numbers of the expression cassette integrated in the genome. The clones that only harbor one copy of the integrated expression cassette are resistant to 100 *μ*g/mL zeocin, while integration of 2, 3, and 4 cassette copies causes resistance of up to 500, 1000, and 2000 *μ*g/mL zeocin [26].

### 3.2. Well-Expressing* Pichia *Clones Are Rapidly Screened by Dot and Colony Blot

**Figure 2:**
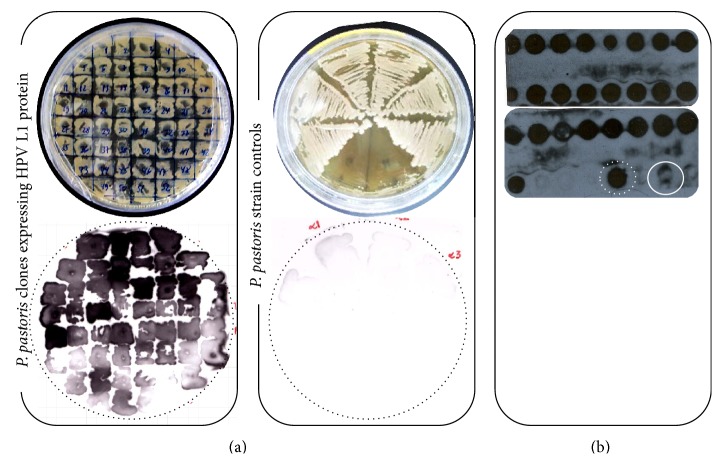
Selection of well-expressing clones.* P. pastoris* transformants were subsequently subjected to higher drug levels (100, 500, and 1000 *μ*g/mL zeocin) for screening of multicopy strains. (a) Secretion of HPV L1 protein from the* P. pastoris*/pPGKΔ3*α*/L1 clones resistant to 1000 *μ*g/mL zeocin was detected by colony blot. Absence of detection in the* P. pastoris* strains controls (yeasts transformed with the parental vectors) ensured the reliability of the reaction. (b) Intracellular expression of HPV L1 protein was confirmed in* P. pastoris*/pPGKΔ3/L1 clones resistant to 1000 *μ*g/mL zeocin by dot blot. HPV16 L1 protein episomally expressed in* P. pastoris* was used as positive control (dotted circle in white), while* P. pastoris* strain transformed with the empty vector was used as negative control (circle in white).

Compared with* E. coli*-based expression systems, the main disadvantage of* P. pastoris* is that it relies on the heterogeneous expression levels of the exogenous gene when the primary transformants are being analyzed [43]. Multicopy screening has the potential to enhance strains expressing increased levels of the heterologous gene, but only a small portion (5%) of highly drug-resistant colonies are generated as a result of an increased gene dosage. Most transformants are resistant to drugs for other (unknown) reasons [13]. Hence, we established a workflow based on the cultivation of recombinant yeasts in deep-well and agar plates and carried out a further analysis by dot and colony blot to identify well-expressing clones. Through this general approach, we were able to access the expression levels of 52 pPGKΔ3*α*/L1H16 colonies and 25 pPGKΔ3/L1H16 colonies. The multicopy clones generated by the PTVA process displayed a uniform expression signal (Figures 2(a) and 2(b)). The lack of detection by the* P. pastoris* cells that had been transformed by the parental expression vectors (empty PGK-based vectors) together with the detection of the L1 protein expressed episomally in* P. pastoris *cytosol ensured the specificity of the reaction.

It is worth noting that this practical and rapid workflow provides a strategy to screen well-expressing clones through cultivation under nonoptimal conditions—with regard to culture volume, shaking, and aeration, as is the case for deep-well and agar plate cultures—among a considerable high number of colonies. The colony blot assay has an important feature, particularly in the case of PGK-based clones that secrete L1 protein, which is the dispensable use of liquid media. Since* P. pastoris* secretes few proteins into the medium [25, 30, 43], screening strategies based on cultivation in liquid media may be unsuitable and require protein precipitation, for example, which is not the case here.

### 3.3. HPV16 L1 Expression in Shake Flasks under* PGK1* Regulation

Expression of the major capsid protein of HPV16 in* P. pastoris* was first reported by Bazan et al. [22]. According to these authors, the expression level achieved was higher than that obtained in* S. cerevisiae*. Despite being innovative and important means of demonstrating the feasibility of* Pichia* in producing HPV VLPs, the work carried out by Bazan and colleagues, employed a nonintegrative system. As the authors (and other studies) point out, the use of episomal plasmids is not recommended for industrial purposes, since they have been associated with the generation of recombinant strains that are genetically unstable and have two undesirable outcomes: (i) instability in the heterologous expression levels; (ii) continuous antibiotic selection which is needed to maintain the expression plasmids [25, 26, 44]. Heterologous L1 protein from other HPV genotypes was also reported in* P. pastoris* by employing AOX1-based integrative vectors [12, 23, 24].

With regard to the metabolization of methanol,* P. pastoris* strains present three distinct phenotypes: (i) the wild-type phenotype designated as methanol utilization plus (Mut^+^) and characterized by the presence of a functional copy of the alcohol oxidase 1 gene (AOX1) which is responsible for 85% of the utilization of methanol by the alcohol oxidase enzyme; (ii) methanol utilization slow (Mut^s^) phenotype, characterized by the absence of the AOX1 gene and presence of the AOX2 gene, which is about 97% homologous to AOX1 but much less expressed; (iii) methanol utilization minus (Mut^−^) phenotype, where both AOX genes are absent. Hence, while Mut^s^ strains show a poor growth on methanol medium, the Mut^+^ strains have a greater growth rate and Mut^−^ strains, in contrast, are unable to grow on methanol as the sole carbon source. Interestingly, Cregg et al. [27] showed that* P. pastoris* Mut^+^strains expressed 10-fold less HBsAg than Mut^s^ strains, which suggests that consumption of methanol at high levels, coupled with a rapid growth rate, may not lead to efficient HBsAg assembly [26]. In addition, HPV VLPs expressed under AOX1 regulation were described as unstable and inadequately assembled [22], although these features were reversed after the incubation of the VLPs under refolding conditions. In light of this, these findings suggest that cell requirements during P_AOX1_ induction are nonoptimal for the production of HBV and HPV VLPs. Finally, even though recombinant protein expression in* Pichia *Mut^s^ strain could circumvent the deficiencies observed for the generation of VLPs in Mut^+^ strains, the slower methanol utilization phenotype requires long fermentation times to reach peak product concentrations. Once induced, the entire culture cannot be used to start a new culture [26].

**Figure 3:**
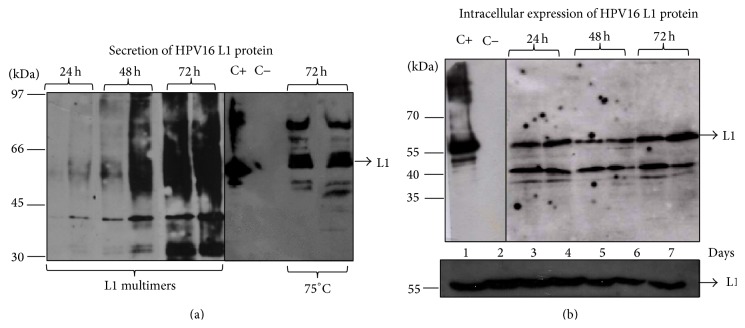
Expression of L1 protein in shake flasks. (a and b) HPV L1 protein was constitutively secreted to the culture media or intracellularly produced after cultivation of four* P. pastoris* clones (two clones for each P_PGK1_-based vector) with glucose for 72 hours. Upon boiling, multimerization of L1 protein in the protein extract was observed as a protein smear, which was overcome after denaturation at 75°C. Aliquots of HPV16 L1 protein episomally expressed in* P. pastoris* [13] were used as a positive control (C+), while the protein extracts from* P. pastoris* strains transformed with the parental vectors were used as negative control (C−).

In contrast with the reports employing P_AOX1_-based system, we constitutively expressed the L1 protein through P_PGK1_ in the presence of glucose and by employing an easier cultivation/expression schedule in shake flasks, since L1 expression occurred together with the cell growth and the laborious control of the methanol/inducer levels was dispensable (Figures 3(a) and 3(b)). By using the anti-HPV16 L1 monoclonal antibody, it was demonstrated that a 56 kDa protein was present in the cellular lysate during the cultivation course (Figure 3(b), L1 secretion will be further discussed), while no detection was observed in the control extracts. A continuous culture strategy, which is a cost-effective method for large-scale production, could be achieved through the use of P_PGK1_-driven expression and would be attractive as it allows an indefinite theoretical production of the heterologous protein [26]. Although there have been a number of different reports showing genetic instability in* P. pastoris* multicopy strains induced with methanol [45–47], we have not observed the same results with the PGK-based clones explored in our work. HPV L1 has been successively detected by western blot after approximately 90 generations, even in the absence of zeocin (Figure 3(b), lower panel). Although more investigations need to be carried out with regard to the structural stability of these clones, these preliminary data suggest there is another advantage related to the use of the* P. pastoris PGK1* promoter.

It has been argued that creating optimum conditions for the production and purification of HPV VLPs is a strategy that can reduce the production costs of vaccines [31, 40], since it could require less time and labor in industrial production. In this regard, different procedures have been explored related to VLP purification steps, such as ultracentrifugation, size-exclusion chromatography, and cation-exchange chromatography or even their combination [39–41], as well as the findings about how cell culture conditions can be optimized [31, 38]. We believe that secretion of HPV L1 in the culture media could improve the downstream process. Yeasts such as* S. cerevisiae* and* P. pastoris* have low specificity requirements for signal sequence recognition [25]. The well characterized* S. cerevisiae α*-MF is the most used secretion signal for* P. pastoris* and achieves similar and even higher expression levels than the* Pichia* native signal sequence [30, 48]. In attempting to obtain secretion of L1 protein at high levels in the culture medium, we employed a codon-optimized *α*-MF along with the PGK1-based vectors. Our initial attempts to detect HPV L1 protein in supernatant of* P. pastoris*/pPGKΔ3*α*/L1H16 clones showed an unexpected protein smear when the samples were boiled at 95°C, which was not visualized in the extract of negative controls (Figure 3(a)). A similar observation was reported earlier [12], probably due to multimerization of L1 protein in higher structures upon boiling. Although this feature was not pronounced when the cell lysate was analyzed, we proceeded with the protein denaturation at 75°C for all the samples before fractioning in electrophoresis, and this allowed the detection of the L1 protein in its expected molecular weight (56 kDa, Figures 3(a) and 3(b)). Lower bands observed in both media and cell lysate-derived samples are possibly degradation products of the L1 protein, since these species were not seen in the negative controls. Similar degradation patterns were also reported previously [12, 24]. When the identical electrophoretic pattern displayed by the secreted L1 protein was compared with both its nonsecreted L1 version and the L1 episomally expressed version, it was suggested the *α*-MF was being processed correctly. In addition, this indicates the lack of posttranslational modification in the secreted L1 protein, although previous reports had characterized glycosylation sites in the HPV1n6 L1 protein [49, 50]. More detailed investigations are needed in this area.

### 3.4. Electron Microscopy Evidence of HPV VLP Assembling within the* P. pastoris* Cells but Not in the Culture Media

The assembly of HBV and HCV core proteins into VLPs has been previously reported in yeast [37, 51]. However, no clear evidence has so far been provided to demonstrate that the HPV VLP self-assembled inside the yeast cell. Electron microscopic characterizations of VLP formation were achieved after downstream processing and purification from the yeast cell lysate, which is also true for the VLPs employed in the current HPV vaccines. The demonstration of intracellular VLP formation in* P. pastoris *opens up perspectives for the development of a live attenuated vaccine, since this yeast has been granted the GRAS (Generally Recognized as Safe) status by the FDA (Food and Drug Administration) and has been recognized as an efficient vehicle for the delivery of viral antigens when administered by an oral and intramuscular route [52]. Furthermore, immunization of animals with live bacteria strains expressing HPV VLP has proved to be effective in the anti-L1 IgG production [20].

**Figure 4:**
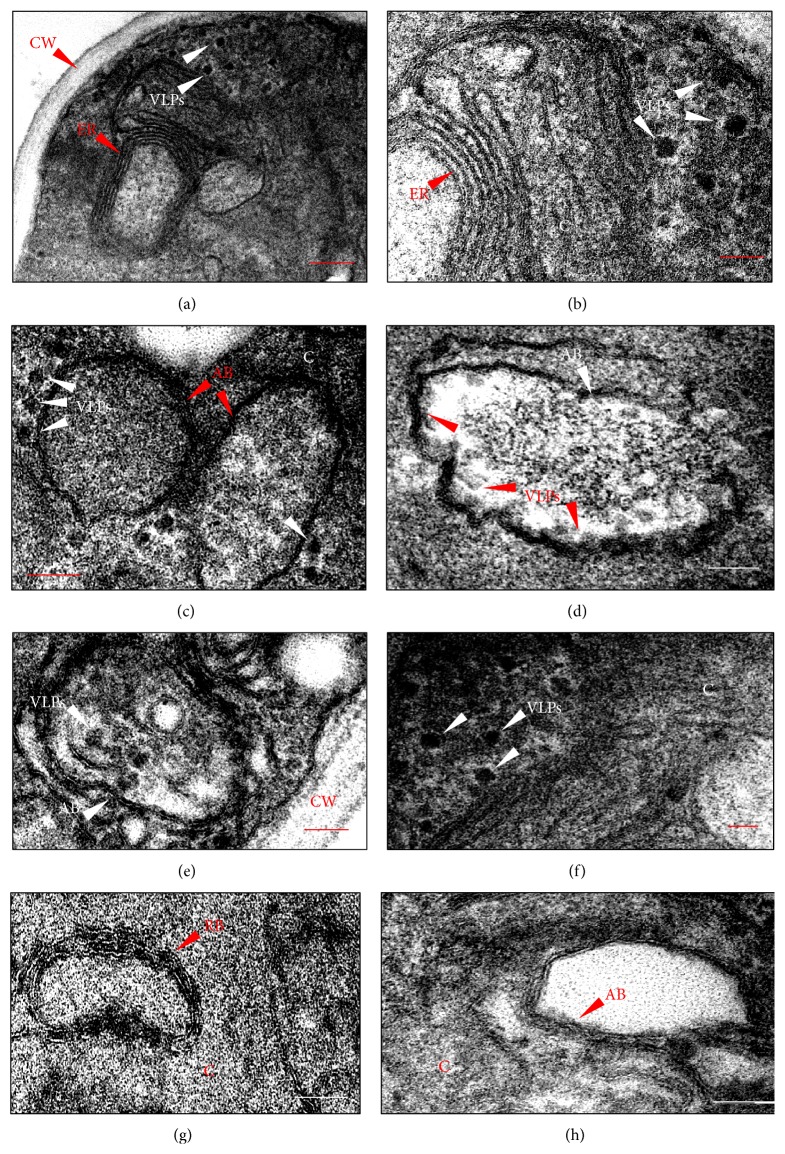
HPV VLPs self-assembly within the* P. pastoris* cells. (a and b) Electrodense structures (VLPs) with estimated diameters of 55 nm were observed near the endoplasmic reticulum (ER) membranes of* P. pastoris* clones expressing L1 protein under regulation of* PGK1* promoter. (c–e) VLPs were visualized both near and inside the autophagic bodies (AB) and were either free from or in contact with the AB membranes. (f) Circumstantially, the VLPs were seen to be transiting in cytoplasm. (g) Close-up of electrodense structures with an estimated size of 20 nm and cluster on ER membranes, which were identified as ribosomes. (h) Similar particles with 55 nm were not observed in the* P. pastoris* cells that were transformed with parental vectors, either in the cytosol or inside AB. (Bar = 200 nm in (a); 80 nm in (b)–(d), (f), and (h); 100 nm in (e); 40 nm in (g)).

Electron microscopy analysis of* P. pastoris* recombinant strains described here provides evidence of electrodense structures with estimated diameters of 55 nm, as expected for the HPV VLPs (Figures 4(a)–4(f)) and in accordance with the western blot analysis that shows the presence of the L1 protein. These particles were located near the endoplasmic reticulum membranes (Figures 4(a) and 4(b)) and inside the autophagic bodies (Figures 4(d) and 4(e)) (either free or apparently interacting with cellular membranes); however, they were absent from the cytoplasm of* P. pastoris* strains that had been transformed by the parental vectors (Figure 4(h)). We also searched for circular, electron dense cellular structures in the cytosol and likewise clustered on membranes which could be mistaken for VLPs. The structures highlighted in Figure 4(g) were identified as ribosomes mainly owing to their small diameter (~20 nm) and differential disposition when attached to membranes and concomitant presence in cells transformed with empty vectors. An immunocytochemical analysis was conducted with a view to providing a further characterization of VLPs within the yeast cells. However, the VLP detection was not possible by means of the CamVir antibody, which is often used to detect HPV16 capsomeres and VLPs [41, 53]. Previous attempts to detect intracellular HCV VLPs in* P. pastoris *through immunoelectron microscopy were hindered by the retention of cell membrane components in the architecture of the HCV core particles which has an envelope-like structure [37]. According to the authors, neither the anti-HCV core monoclonal antibody nor the core-reacting human sera were able to stain the particles, in spite of their visualization by conventional electron microscopy. This data could explain why we could not detect the HPV VLPs.

We attempted to characterize assembled HPV VLPs in the culture media from* P. pastoris* recombinant strains. It has been shown that assembly of HPV VLPs can be achieved with neutral pH, high ionic strength, and relatively low concentrations of reducing agents [54]. Conversely, high pH, low salt concentration, and the presence of reducing agents disassemble the VLP into capsomeres. As* P. pastoris* growth progresses in unbuffered medium, the pH drops to 3 or below [55]. It can be speculated that under this condition, culture media offers nonoptimal conditions for HPV VLPs assembly, since HPV L1 protein was detected by western blot after concentration. Nevertheless, the absence of VLP assembly under our experimental conditions does not compromise the use of L1 secreted protein as immunogens. Particle assembly protocols have been used for HPV VLP formation even when the L1 protein is expressed intracellularly, either to increase production yields or improve particle immunogenicity [22, 48–51]. Since our main objective was to evaluate the feasibility of the system, we believe that dot blot, colony blot, and western blot data ensure that our goal can be achieved.

## 4. Conclusion

The data presented in this work demonstrate the functionality of a biotechnology platform based on* P. pastoris PGK1* promoter for the production of HPV16 VLPs. Constitutive expression of the HPV16 L1 capsid protein was efficiently achieved for the first time through an easier production schedule than is the case when an* AOX1* inducible promoter is employed. In addition, an optimized *α*-MF secretion signal downstream to P_PGK1_ provided a prompt secretion of L1 protein in the culture media. Although further experiments are needed to determine which production strategy is more effective for HPV L1 protein production, the data outlined here underline the efficiency of a PGK1-based platform as a heterologous expression system and support its employment for the production of HPV VLPs for vaccination purposes. Moreover, our preliminary data showing intracellular VLP assembly open up perspectives for the employment of* P. pastoris* cells that can express HPV L1 protein as a live attenuated vaccine. To the best of our knowledge, this is the first time that the expression of HPV L1 protein was achieved in* P. pastoris* through an IP-free biotechnology platform. The discovery of alternatives to biotechnology platforms for producing an efficient and cost-effective vaccine has the potential both to offer greater protection—since not all the HPV genotypes are covered by the current vaccines—and to allow the vaccine to be widely disseminated.
